# Advanced Nucleic Acid Architectures: Classification, Assembly Mechanisms, and Biomedical Applications

**DOI:** 10.3390/pharmaceutics18101337

**Published:** 2026-10-21

**Authors:** Yan Liu, Caiyun Zhao, Chengke Zhao, Siying Li, Chang Xu, Han Wu, Xiaojing Pei, Hong Meng

**Affiliations:** School of Light Industry Science and Engineering, Beijing Technology and Business University, Beijing 100048, China

**Keywords:** nucleic acid architectures, DNA nanotechnology, self-assembly, drug delivery, regenerative biomaterials

## Abstract

Nucleic acids are increasingly exploited as programmable molecular materials for nanomedicine, biosensing, and biomaterials engineering, but their biomedical deployment remains constrained by nuclease degradation, inefficient membrane transport, endosomal sequestration, rapid clearance, and potential immune activation. This Review presents a role-based framework for advanced nucleic acid architectures according to the contribution of nucleic acids to final structure, spanning nucleic acid-dictated architectures, supramolecular nucleic acid co-assemblies, and nucleic acid-loaded composite biomaterials, including sequence-defined genetic cargos and non-sequence-defined PDRN-based regenerative systems. We compare sequence-directed hybridization, electrostatic condensation, metal–ligand coordination, polyamine- and polyphenol-mediated interactions, and carrier- or matrix-confined assembly, and relate these mechanisms to structural stability, nuclease resistance, cellular transport, intracellular release, stimulus responsiveness, and biological activity. Applications in drug and nucleic acid delivery, molecular diagnostics, regenerative and skin-repair biomaterials, vaccination, and immunomodulation are critically evaluated. Across platforms, architecture emerges as a formulation variable that must balance molecular protection with functional release, productive delivery, biosafety, and manufacturability. Translational development requires quantitative structure–performance relationships, standardized characterization, stability assessment, scalable processing, and context-specific pharmacokinetic and regulatory evaluation. This integrated perspective provides design principles for the rational pharmaceutical development of advanced nucleic acid systems.

## 1. Introduction

Nucleic acids represent a fundamental category of biological macromolecules, with deoxyribonucleic acid (DNA) and ribonucleic acid (RNA) being two major types. These molecules are indispensable not only in regulating genetic expression and facilitating protein synthesis, but also in supporting a diverse array of cellular physiological activities [1]. DNA is mainly responsible for storing and transmitting genetic information, and its double helix structure enables stable storage and replication of information through base pairing and base stacking [2]. Ribonucleic acids (RNAs) perform essential functions during the synthesis of proteins, a fundamental biological process. These RNA molecules contribute to the transmission and control of genetic information via distinct mechanisms. Each type of RNA plays a specialized role in ensuring the accurate and efficient expression of genetic material. Messenger RNA, transfer RNA, and ribosomal RNA function as the information template, adaptor molecule, and structural–catalytic core of the translational machinery, respectively [3]. At the molecular level, DNA and RNA are linear polynucleotides assembled from nucleotide monomers, each comprising a nitrogenous base, a pentose sugar, and a phosphate moiety. DNA contains 2-deoxyribose and predominantly thymine, whereas RNA contains ribose and uracil [4]. Nucleotide residues are connected through 3′–5′ phosphodiester linkages to form a chemically regular and negatively charged sugar–phosphate backbone, while the nucleobases mediate sequence-dependent hydrogen bonding and stacking interactions [4,5,6]. This combination of backbone regularity and base-specific molecular recognition provides the structural basis for predictable nucleic acid assembly and site-selective chemical functionalization. Consequently, these intrinsic molecular features establish nucleic acids not only as indispensable biological macromolecules but also as structurally predictable and chemically addressable building blocks for advanced biomaterial design. Beyond canonical DNA and RNA, nucleic acid-derived therapeutic components have attracted increasing attention in regenerative medicine and dermatological repair. Among them, polydeoxyribonucleotide (PDRN) refers to a biologically active mixture of DNA fragments composed of deoxyribonucleotide chains of variable lengths [7]. With molecular weights ranging from approximately 50 to 1500 kDa, these polynucleotide components are typically derived from highly purified sources such as salmon sperm, achieving purities exceeding 95% [8]. PDRN has attracted substantial scientific interest owing to its diverse therapeutic potential in regenerative medicine, dermatology, and anti-inflammatory intervention. Mechanistically, its bioactivity is primarily mediated through a dual pathway: activation of the adenosine A2A receptor [9]—which triggers downstream signaling cascades—and provision of nucleotide precursors that support tissue repair processes [9]. This integrated mode of action underlies its reported biological effects, which include promoting tissue regeneration, mitigating inflammation, exerting antioxidant activity, and enhancing cellular resilience [9]. Importantly, PDRN is conceptually distinct from sequence-defined therapeutic nucleic acids and programmable nucleic acid nanostructures. Whereas antisense oligonucleotides and siRNA depend on sequence-specific molecular recognition, mRNA and plasmid DNA convey sequence-encoded genetic information, and programmable DNA nanostructures rely on sequence-directed assembly, PDRN comprises a heterogeneous population of DNA fragments without a predefined therapeutic sequence. Its regenerative activity is therefore considered largely sequence-independent and is primarily associated with adenosine A2A receptor signaling and nucleotide salvage rather than target-specific gene regulation or sequence-programmed structural information [10].

The conceptual transformation of nucleic acids from genetic molecules into programmable nanomaterials has reshaped modern nanobiotechnology. The predictable Watson–Crick base-pairing rules, combined with controllable strand hybridization, strand displacement, and chemical modification, enable the bottom-up construction of nanoscale structures with defined geometry, addressable sites, and tunable dynamic behavior [11,12,13]. This design principle has led to the emergence of structural DNA nanotechnology, in which DNA is used not only as an information-bearing molecule but also as a molecular construction material for engineering two-dimensional and three-dimensional architectures [14]. These molecular features provide a rational basis for the bottom-up construction of nanostructures with predefined geometry, controllable topology, spatially addressable sites, and stimulus-responsive behavior [15]. Building upon these fundamental properties, recent advances in nucleic acid nanotechnology have expanded the development of dynamically reconfigurable architectures with programmable structures and functional responses. Representative examples include DNA origami nanostructures, framework nucleic acids, spherical nucleic acids, DNA hydrogels, and metal–nucleic acid assemblies. DNA origami provides a powerful route for folding long single-stranded DNA scaffolds into precisely defined nanostructures using short staple strands [15,16]. Framework nucleic acids, particularly tFNAs, provide structurally programmable platforms for cellular uptake, molecular delivery, and biomedical modulation [17]. Spherical nucleic acids present densely oriented nucleic acid shells on nanoparticle or molecular cores, thereby generating architecture-dependent biological properties distinct from linear oligonucleotides [18]. Together, these systems demonstrate that nucleic acids can encode not only biological information but also structural form, spatial organization, molecular recognition, and therapeutic function.

Despite their remarkable programmability, nucleic acids still face intrinsic barriers to biomedical translation, including nuclease degradation, poor membrane permeability, endosomal entrapment, rapid clearance, and potential immunostimulation [19,20]. To address these limitations, advanced nucleic acid systems increasingly integrate chemical modifications, electrostatic condensation, metal coordination, polycationic complexation, polyphenol adhesion, hydrogel crosslinking, and lipid or polymeric nanocarrier confinement [21]. These strategies improve stability, enhance cellular internalization, regulate release kinetics, and expand the therapeutic potential of nucleic acid-based systems. Researchers have successively developed a variety of innovative structural systems based on nucleic acid advanced organization structures, which realize the adaptive needs of diverse application scenarios by precisely regulating the molecular assembly method. These engineered nucleic acid nanostructures have been investigated in multiple biomedical applications, including drug delivery, gene regulation, and regenerative medicine. In drug delivery systems, these sophisticated architectures can safeguard nucleic acid therapeutics against enzymatic degradation while enhancing bioavailability and enable targeted delivery through precise structural modifications. Depending on the intended application, delivery performance should be evaluated at distinct biological barriers. For cutaneous formulations, relevant endpoints include penetration across the stratum corneum, deposition within viable skin layers, local retention, and cargo release. For gene-active nucleic acids, cellular internalization represents only an initial step and should be distinguished from endosomal escape, cytosolic or nuclear cargo availability, and the resulting functional activity [22].

Recent reviews have advanced the field of nucleic acid nanotechnology from complementary but largely domain-specific perspectives. Ma et al. [23] summarized the structural development and biomedical applications of DNA nanomaterials, whereas Chang et al. [24] reviewed DNA self-assembly strategies and designer nucleic acid nanostructures for biomedical applications. Other recent reviews have focused on the clinical translation of nucleic acid nanoparticles, DNA-directed nanomaterial assembly, nanocarrier-mediated nucleic acid delivery, DNA/RNA origami, or function-oriented DNA nanotechnology [25]. Collectively, these studies have provided important insights into specific material classes, assembly paradigms, delivery platforms, and translational issues [26]. Although existing classifications have substantially advanced the understanding of nucleic acid-based systems, they primarily organize these platforms according to material composition, structural morphology, or biomedical application. Such approaches are valuable for describing individual systems but provide limited insight into how the functional role of nucleic acids during assembly influences the resulting architecture and biological behavior. Therefore, we propose a complementary role-based framework that categorizes advanced nucleic acid architectures according to their structural dependence on nucleic acids. This perspective facilitates mechanistic analysis by addressing three fundamental questions: whether nucleic acids serve as the primary architectural blueprint, whether they cooperate with external components to establish supramolecular structures, or whether they function mainly as therapeutic or bioactive cargos within carrier-dominated systems. By linking structural contribution with assembly mechanism and functional outcome, this framework enables more systematic comparison of diverse nucleic acid platforms beyond conventional material- or application-based classifications.

Accordingly, this Review classifies advanced nucleic acid architectures according to their dominant structural determinants rather than the presence of specific material components. Nucleic acid-dictated architectures are governed primarily by sequence-programmed organization, topology, or nucleic acid-mediated network formation. Supramolecular nucleic acid co-assemblies are established through cooperative interactions between nucleic acids and external molecular partners, whereas nucleic acid-loaded composite biomaterials are mainly determined by carrier or matrix structures with nucleic acids serving functional roles. Because many emerging platforms exhibit overlapping structural features, these categories should be considered as a continuum rather than strictly discrete classes. The operational criteria for classification are summarized in Table 1. Accordingly, the inclusion of PDRN in this framework reflects its role as a DNA-derived bioactive cargo within material assemblies and should not be interpreted as mechanistic equivalence to sequence-defined nucleic acid therapeutics.

Within this framework, the present Review moves beyond a descriptive catalog of individual nucleic acid systems to examine how distinct molecular interactions and assembly processes translate into physicochemical properties and biological performance. Particular attention is given to the relationships between sequence-directed hybridization, electrostatic condensation, metal coordination, polyphenol-mediated interactions, and carrier confinement and key functional outcomes, including structural stability, nuclease resistance, cellular transport, intracellular release, stimulus responsiveness, and biological activity [16,27,28]. These relationships are further examined across drug and gene delivery, molecular diagnostics, regenerative and skin-repair biomaterials, vaccination, and immunomodulation. Finally, we critically discuss the major design trade-offs and translational constraints shared across these systems, including biosafety, structural reproducibility, manufacturing scalability, quality control, and regulatory considerations, with the aim of identifying general principles for the rational design and biomedical translation of advanced nucleic acid architectures.

**Table 1:** Role-based classification and structural contribution criteria of advanced nucleic acid architectures.

Class	Primary Structural Determinant	Role of Nucleic Acid	Representative Architectures	Operational Classification Criterion
Nucleic acid-dictated architectures [15,18,29,30]	Sequence-programmed hybridization, nucleic acid topology, radial nucleic acid arrangement, or nucleic acid-mediated network formation as the dominant structural contributors	Major structural component responsible for defining geometry, spatial organization, or molecular addressability	DNA origami; tetrahedral framework nucleic acids (tFNAs); spherical nucleic acids (SNAs); DNA hydrogels;	Removal or disruption of nucleic acid organization substantially alters the defining architecture or functional interface
Supramolecular nucleic acid co-assemblies [31,32,33,34,35,36,37]	Cooperative noncovalent interactions between nucleic acids and external components, including electrostatic association, metal coordination, hydrogen bonding, or molecular adhesion	Structural contributor that cooperates with external components to regulate morphology, stability, and dynamic behavior	Chitosan–DNA/PDRN; polyamine–DNA condensates; Fe–DNA nanospheres; Cu–DNAzyme nanohybrids; DNA–tannic acid assemblies	Final architecture requires a combined contribution from nucleic acids and external components, although their relative structural contributions may vary among systems
Nucleic acid-loaded composite biomaterials [38,39,40,41]	Carrier or matrix assembly processes, including polymer networking, lipid self-assembly, or hydrogel formation	Functional component primarily contributing biological activity rather than determining bulk architecture	PLGA/PEI–pDNA microparticles; lipid nanoparticles; alginate–PDRN and hyaluronic acid–PDRN hydrogels;	Carrier or matrix properties remain the dominant determinants of overall architecture after considering nucleic acid contributions
Hybrid/borderline architectures [42,43]	Comparable or context-dependent contributions from nucleic acids and non-nucleic-acid components	Both structural and functional contributions that cannot be separated into a single dominant category	Core–shell SNAs, lipid-associated nucleic acid assemblies, DNA/polymer hybrid hydrogels	Assigned according to the experimentally dominant structural contributor under the specific assembly conditions

Abbreviations: PDRN, polydeoxyribonucleotide; PLGA, poly(lactic-co-glycolic acid); PEI, polyethylenimine; pDNA, plasmid DNA. Note: This classification is based on the structural role of the nucleic acid component rather than pharmacological equivalence among nucleic acid modalities. PDRN represents a heterogeneous, non-sequence-defined DNA-derived bioactive, whereas siRNA, mRNA, and pDNA are sequence-defined genetic cargos.

### Literature Search Strategy

This Review was prepared as a structured narrative synthesis. The relevant literature was identified through PubMed, Web of Science Core Collection, and Scopus, with Google Scholar used for supplementary citation searching. The search primarily covered January 2000 to September 2026; earlier landmark studies were retained when they established foundational concepts relevant to nucleic acid assembly. Search terms were combined using Boolean operators and organized into four concept domains: (i) nucleic acid architectures, including DNA origami, framework nucleic acids, spherical nucleic acids, and DNA hydrogels; (ii) supramolecular assembly mechanisms, including electrostatic condensation, polycation/polyamine complexation, metal coordination, polyphenol-mediated interactions, and surfactant-assisted assembly; (iii) carrier- or matrix-dominated systems, including lipid nanoparticles, polymeric carriers, PDRN/polydeoxyribonucleotide, alginate, and hyaluronic acid hydrogels; and (iv) biomedical functions, including drug or gene delivery, biosensing, skin and wound repair, vaccination, and immunomodulation. Peer-reviewed original studies and review articles were considered. Reviews were used primarily for field mapping and citation tracking, whereas mechanistic statements were preferentially supported by primary studies. Study selection prioritized seminal reports, direct experimental evidence linking assembly or structural attributes to functional outcomes, translational relevance, and balanced representation across the structural categories considered in this Review. When multiple studies supported the same conclusion, the seminal report and/or a recent study providing direct mechanistic or quantitative evidence was preferentially cited. Studies lacking a relevant engineered nucleic acid architecture or material-level mechanistic relevance, duplicate reports, conference abstracts, and non-peer-reviewed records were not considered. The resulting literature set was intended to provide representative rather than exhaustive coverage of the field.

## 2. Structural Categories of Advanced Nucleic Acid Architectures

Advanced nucleic acid architectures are classified here according to the relative contribution of nucleic acids and non-nucleic-acid components in determining the final structure. Nucleic acid-dictated architectures are primarily defined by nucleic acid-programmed geometry, spatial organization, or network formation; supramolecular nucleic acid co-assemblies arise from the cooperative contribution of nucleic acids and external molecular or ionic partners; and nucleic acid-loaded composite biomaterials are predominantly shaped by carrier or matrix components, with nucleic acids functioning mainly as therapeutic, gene-regulatory, or regenerative elements. This role-based classification provides a consistent framework for comparing their assembly mechanisms and structure–function relationships. However, several advanced nucleic acid systems exhibit overlapping characteristics because nucleic acid organization and external components can simultaneously contribute to architecture formation. These hybrid or borderline architectures are therefore discussed according to the predominant contributor governing their overall architecture under specific assembly conditions rather than being considered as strictly discrete classes [15,44,45,46]. Viewed as a continuum, these three categories represent a progressive shift in the structural role of nucleic acids: from the dominant architecture-defining scaffold, through a cooperative co-structuring component, to a primarily functional cargo or bioactive component within a carrier- or matrix-dictated architecture.

### 2.1. Nucleic Acid-Dictated Architectures

#### 2.1.1. Spherical Nucleic Acids

**Figure 1:**
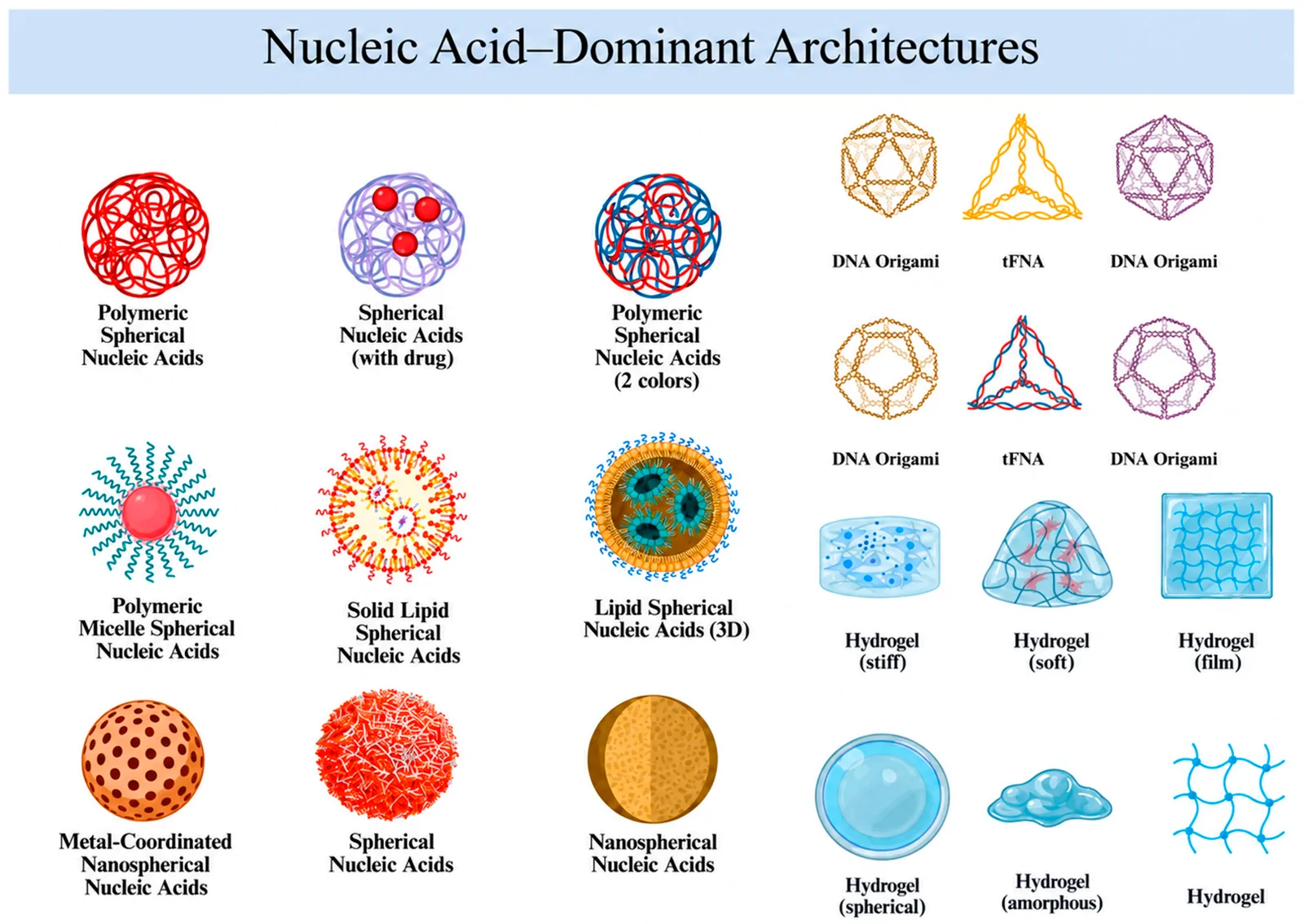
Representative nucleic acid-dictated architectures, in which nucleic acid organization acts as the dominant structural determinant.

The process of nucleic acid spherization generates architecturally spherical nucleic acid nanostructures through controlled molecular assembly [47]. This structural configuration is typically achieved by the dense, organized arrangement of nucleic acid strands on nanoscale substrates, including, but not limited to, lipid-based RNA nanoparticles (Figure 1). Selected SNA formulations have shown greater nuclease resistance and cellular internalization than free oligonucleotides, with uptake depending on core composition, oligonucleotide density, surface chemistry, and cell type. These findings support architecture-dependent endocytic uptake rather than passive membrane-barrier bypass; productive delivery additionally requires appropriate intracellular trafficking and cargo release [48]. For example, spherical nucleic acids (SNAs) exhibit remarkable functional versatility in transporting therapeutic nucleic acids. SNAs can enhance the cellular internalization of nucleic acid cargos relative to free oligonucleotides; however, internalization alone does not establish productive intracellular delivery. Functional delivery additionally depends on intracellular trafficking, cargo release, and, where required, access to the cytosol or nucleus [49]. The comparative design features of nucleic acid regulatory structures have been summarised in Table 2.

#### 2.1.2. DNA Origami Nanostructures

DNA origami techniques enable the precise construction of diverse nanostructures with programmable two-dimensional (planar), three-dimensional (solid), or hollow geometries [15] (Figure 1). As a representative example, Peil et al. demonstrated an innovative approach inspired by transition metal-templated streptavidin synthesis, wherein they engineered a long single-stranded M13 DNA scaffold through hybridization with approximately 210 staple strands to form quarter-ring origami monomers [50]. Each monomeric unit comprises 24 precisely arranged DNA helices featuring two specific binding domains at the termini. Through the programmed assembly of four such monomers, the researchers successfully constructed a DNA origami ring structure with a well-defined inner diameter of approximately 120 nm. This example illustrates how staple connectivity, crossover positioning, and hierarchical assembly can encode controlled curvature and higher-order topology.

The structural programmability of DNA origami can further be extended to nanopore engineering. DNA origami has been integrated with solid-state nanopores or engineered as membrane-spanning channels, allowing pore dimensions, chemical functionalities, and membrane-anchoring motifs to be defined with nanometer-scale precision. Such architectures provide a programmable structural basis for controlled molecular translocation and single-molecule ionic-current sensing [51,52,53,54]. Their sensing mechanisms and analytical applications are discussed further in Section 4.3.

Although DNA origami nanostructures are commonly described based on their programmed geometries and staple-mediated folding strategies, their behavior in practical environments is governed by a more complex interplay of electrostatic interactions, hydration effects, and mechanical adaptability. Therefore, DNA origami assemblies should be considered dynamic polyelectrolyte systems rather than rigid nanoscale frameworks. In particular, their structural stability is highly dependent on ionic composition, solvent conditions, and external physical constraints. Although Mg^2+^-rich buffers are widely used during DNA origami assembly, the stability of these structures under physiological or low-Mg^2+^ conditions cannot be directly predicted from their assembly-state morphology. Variations in divalent cation concentration, buffer composition, and origami architecture can significantly influence helix–helix interactions, crossover stabilization, and overall structural integrity [55]. Molecular simulations have provided important insights into the physicochemical mechanisms underlying DNA origami stability and deformation. Atomistic molecular dynamics simulations revealed that counterions regulate origami stability by modulating electrostatic repulsion between neighboring DNA helices and maintaining crossover conformations, with Mg^2+^ and polyamines exhibiting distinct stabilization mechanisms compared with monovalent ions [56]. At larger spatial and temporal scales, computational approaches such as CanDo and coarse-grained models including oxDNA enable quantitative analysis of equilibrium structures, thermal fluctuations, bending rigidity, twisting behavior, and conformational transitions of DNA origami assemblies [57]. Furthermore, free-energy-based simulations and mechanical modeling have demonstrated that environmental constraints, including molecular confinement and external forces, can reshape the conformational landscape of dynamic DNA origami systems. Such mechanically responsive behavior is particularly relevant for biomedical applications, where DNA origami structures encounter complex biological environments involving steric confinement, heterogeneous ionic conditions, and fluidic forces [58].

Collectively, these studies highlight that the functional performance of DNA origami is determined not only by its designed nanoscale geometry but also by its ability to maintain structural integrity and adapt mechanically under application-relevant conditions. Future evaluations of DNA origami-based systems should therefore incorporate environmental factors, molecular flexibility, and computationally predicted structural responses rather than relying solely on static structural characterization. Recent work has shifted DNA origami from structural proof-of-concept toward in vivo functional validation. Trigger-responsive origami nanodevices have enabled conditional therapeutic exposure in animal models, whereas current translational analyses identify biological-barrier crossing, nonspecific uptake, immune recognition, quantitative delivery efficiency, and scalable manufacture as key requirements for further clinical development [59,60].

#### 2.1.3. Framework Nucleic Acids

A representative example of framework nucleic acids is the tetrahedral framework nucleic acid architecture (tFNA), a DNA-based nanostructure assembled through programmed interactions among four oligonucleotide strands. The tetrahedral architecture is generated through sequence-specific Watson–Crick base pairing among four DNA strands, enabling the construction of a defined three-dimensional framework (Figure 1) [17,43]. However, the tetrahedral motif defines the nominal geometry rather than a fixed hydrodynamic size. Reported tFNA diameters vary among formulations, and cargo incorporation can further alter particle dimensions, indicating that particle size should be treated as a formulation-dependent attribute rather than an intrinsic constant of the architecture. tFNA has been investigated in various biomedical contexts, including drug delivery, tissue regeneration, and molecular sensing. Its structural features allow sequence-based modification and incorporation of functional molecules, providing a platform for regulating biological interactions and delivering therapeutic cargos. Compared with conventional linear nucleic acids, the compact three-dimensional architecture of tFNA contributes to improved structural stability and facilitates cellular internalization. Although DNA molecules possess an intrinsic negative charge that generally limits membrane transport, tFNA can enter cells through caveolin-dependent endocytic pathways without additional transfection reagents [29,61,62]. Across different biological models, enhanced internalization of tFNA relative to linear ssDNA is a recurring observation, whereas the downstream biological response is more context-dependent. For example, tFNA-associated responses have involved p38/ERK1/2 signaling in corneal epithelial cells and Wnt-related signaling in osteoclast models [63]. Importantly, fluorescence-based intracellular accumulation should not be interpreted as direct evidence of productive cytosolic delivery; endosomal escape therefore requires separate experimental verification. In selected preclinical models, tFNA formulations have shown low cytotoxicity and limited immune activation relative to unstructured nucleic acids [29]. These characteristics have supported the exploration of tFNA-based systems in biological regulation, drug delivery, and regenerative medicine. However, challenges including large-scale fabrication, long-term in vivo stability, and clinical translation require further investigation.

Wogonin is a flavonoid compound reported to possess antioxidant and anti-inflammatory activities [64,65]. tFNA-based nanostructures have been used as carriers for wogonin delivery, forming composite tFNA/wogonin systems. These complexes demonstrated anti-inflammatory and chondroprotective effects in both cell culture and animal models [66]. In this representative formulation, cargo loading increased the hydrodynamic size from approximately 7 nm for tFNA to approximately 20 nm for the tFNA/wogonin complex, illustrating that functionalization can alter the physicochemical attributes of the carrier rather than simply add therapeutic activity [67]. The integration of wogonin with tFNA provides a delivery approach that combines the biological activity of the small molecule with the structural properties of DNA frameworks, including cellular uptake and cargo delivery capability. Accordingly, loading efficiency and cargo-induced changes in particle dimensions should be evaluated together with stability, intracellular release, and biological activity. Further studies are required to optimize formulation parameters, quantify cargo release and cytosolic availability, determine how formulation-dependent changes in particle properties influence in vivo disposition, evaluate long-term biosafety, and determine translational feasibility. Recent translational assessments further emphasize that tFNA development requires the batch-level control of strand stoichiometry, assembly yield, structural integrity in biological fluids, pharmacokinetics, and route-dependent biodistribution. These parameters should be evaluated together with functional delivery and long-term safety before clinical extrapolation [68].

#### 2.1.4. DNA Hydrogels

DNA hydrogels are a class of advanced biomaterials constructed from DNA molecules, which form 3D networks through self-assembly or cross-linking mechanisms (Figure 1). The rational design of these hydrogel systems is fundamentally based on the characteristic behaviors of DNA, particularly its capacity for specific molecular recognition through Watson–Crick interactions. This programmability allows for the precise spatial organization of constituent polymers, resulting in networks with highly customizable physical and chemical properties [30]; The other is to form a gel by enzymatically ligating DNA scaffolding structures [69]. A primary classification of DNA-based hydrogels distinguishes two fundamental types based on their composition. The first category integrates synthetic polymer chains, utilizing nucleic acid sequences principally as structural crosslinkers to form composite networks. In contrast, the second type consists exclusively of DNA molecules, which self-assemble into hydrogel matrices through programmed molecular interactions [43]. The exceptional molecular programmability and structural adaptability of DNA hydrogels position them as a transformative platform in biomedical applications. Their precisely tunable physicochemical properties enable diverse implementations in regenerative medicine, controlled therapeutic delivery systems, and advanced biomedical instrumentation [37]. These characteristics support further investigation of DNA-based hydrogels in controlled delivery and regenerative medicine applications.

**Table 2:** Comparative design features of nucleic acid-dictated architectures.

Architecture	Assembly Logic	Typical Topology	Programmable Attributes	Distinctive Functional Advantage
Spherical nucleic acids (SNAs) [18,47,49]	Dense radial presentation of oligonucleotides on a nanoparticle or self-assembled core	Spherical core–shell nanoarchitecture	Sequence, shell density, orientation, core identity, and targeting ligand density	Architecture-dependent cellular uptake, nuclease resistance, and multivalent recognition
DNA origami [15,50,51,52,53,54,58,70]	Folding of a long scaffold by sequence-specific staple strands	2D sheets, 3D solids, hollow objects, rings, reconfigurable devices, and membrane-spanning or hybrid nanopores	Shape, dimensions, addressable sites, pore diameter, channel length, membrane anchors, cargo spacing, and dynamic/gating elements	Nanometer-scale spatial organization and programmable molecular transport, enabling geometry- and chemistry-defined single-molecule sensing and controlled delivery
Tetrahedral framework nucleic acids (tFNAs) [71,72,73]	Programmed hybridization of four oligonucleotides into a closed tetrahedron	Rigid tetrahedral nanoframework	Edge length, vertex modification, cargo attachment, and sequence composition	Enhanced cellular uptake reported in selected models without conventional transfection reagents; biocompatibility remains context-dependent
DNA hydrogels [30,43]	Hybridization-mediated crosslinking, enzymatic ligation, or DNA-only network assembly	Three-dimensional hydrated network	Crosslink density, mesh size, degradability, and thermal/enzymatic responsiveness	High local loading, tissue retention, and programmable release

This table emphasizes nonredundant design variables and key limitations; individual experimental examples may remain in the corresponding subsections.

## 3. Mechanisms of Supramolecular Nucleic Acid Co-Assemblies

Throughout this section, mechanistic assignments are described as established only when supported by direct experimental evidence; otherwise, relationships are identified as proposed or correlative. The formation of higher-order nucleic acid architectures is governed by a combination of noncovalent interactions, primarily electrostatic attraction, hydrogen bonding, and base-stacking interactions. The negatively charged phosphodiester backbone of DNA and RNA promotes association with cationic species, including multivalent ions, polyamines, cationic polymers, and lipid-based cations, thereby facilitating charge neutralization, molecular condensation, and supramolecular assembly. Such electrostatic interactions play a central role in nucleic acid compaction and in the stabilization of nucleic acid–cation complexes [74]. Hydrogen bonding provides sequence-specific molecular recognition through complementary base pairing and is essential for the formation of DNA duplexes as well as the secondary and tertiary folding of RNA [75]. In parallel, base-stacking interactions between adjacent nucleobases further stabilize nucleic acid conformations through aromatic stacking and associated dispersion and hydrophobic contributions. Together, these noncovalent interactions provide the physicochemical basis for controlling the assembly, stability, morphology, and functional behavior of nucleic acid-based supramolecular systems [76]. A summary of the classifications is provided in Table 3.

### 3.1. Electrostatic Complexation

DNA and RNA are polyanionic macromolecules owing to the negatively charged phosphate groups in their backbones. Electrostatic repulsion between adjacent nucleic acid segments generally favors an extended conformation, whereas association with multivalent cations, polyamines, cationic polymers, or cationic lipids reduces the effective negative charge and promotes chain collapse. Under appropriate charge ratios and ionic conditions, both DNA and RNA can therefore be condensed into compact globular, spherical, or toroidal assemblies [77]. Poly-L-lysine, for example, cooperatively condensed plasmid DNA into spherical particles with diameters of approximately 15–30 nm, whereas electrostatic complexation of double-stranded RNA with chitosan–tripolyphosphate generated positively charged spherical nanoparticles smaller than 200 nm [78,79]. The morphology and colloidal stability of these condensates are governed by nucleic acid length and conformational rigidity, cation valency and charge density, the cation-to-phosphate ratio, ionic strength, and the sequence of component mixing [32]. Electrostatic condensation reduces the effective dimensions of nucleic acids and can enhance nuclease protection and cellular internalization, thereby providing a general physicochemical basis for constructing nonviral nucleic acid delivery systems. Nevertheless, excessively strong complexation may impede intracellular nucleic acid release, indicating that condensation stability and cargo accessibility must be carefully balanced.

#### 3.1.1. Chitosan

**Figure 2:**
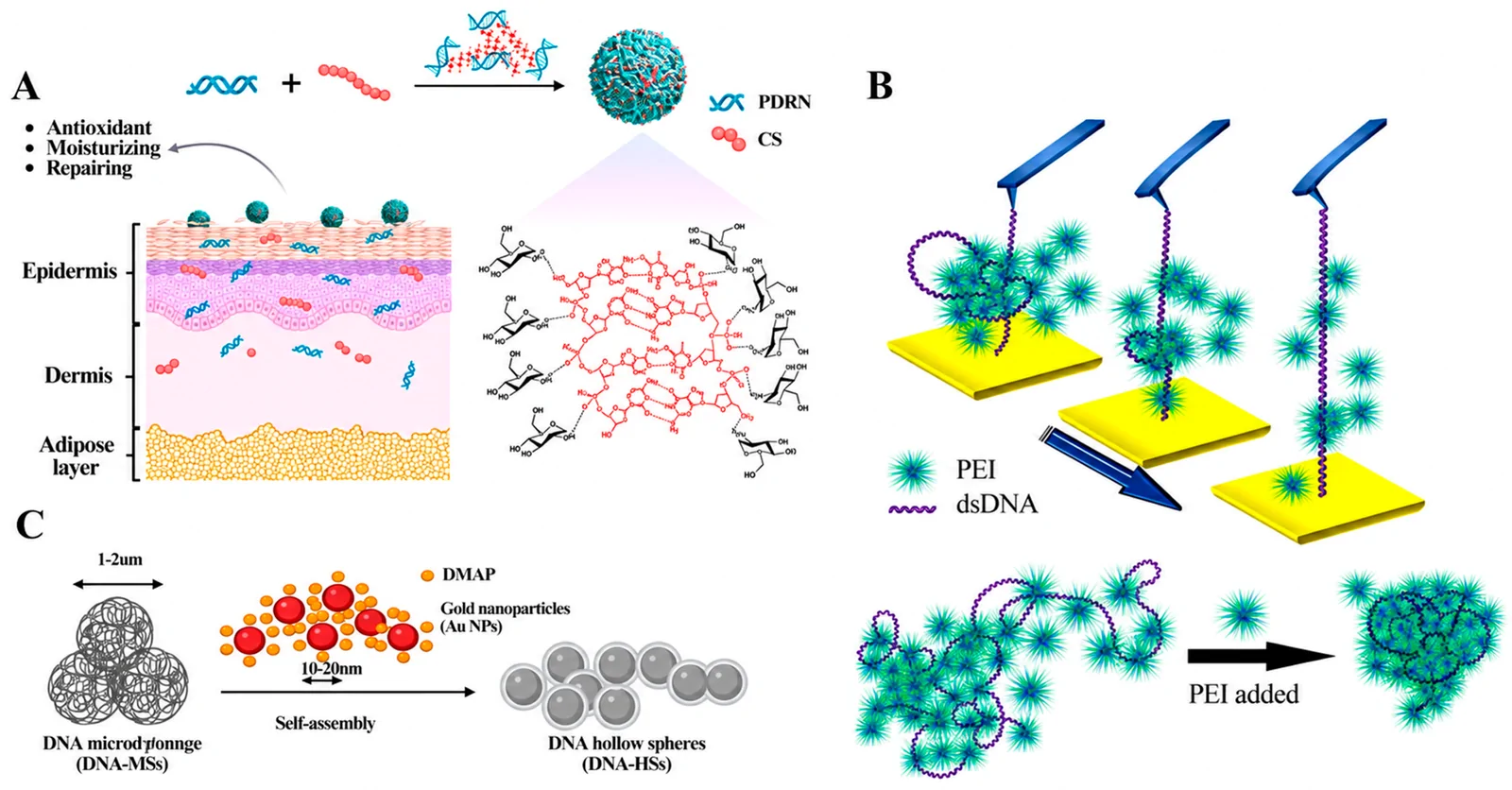
Representative electrostatically driven nucleic acid co-assemblies, in which nucleic acids and cationic partners jointly determine nanoscale organization. (**A**) CP-NPs. Reprinted with permission from ref. [31], Copyright (2026) *European Polymer Journal*. (**B**) PEI–DNA. Reprinted with permission from ref. [93], Copyright (2016) *International Journal of ACS Applied Materials & Interface*. (**C**) DNA-HSs.

Chitosan forms nucleic acid polyplexes through electrostatic association between protonated primary amines and anionic phosphate groups, while its biodegradability and mucoadhesive character support nonviral delivery [64,65,66,67,68]. For chitosan specifically, molecular weight (MW), degree of deacetylation (DDA), degree of protonation, and N/P ratio jointly determine particle dimensions, surface charge, colloidal stability, and nucleic acid binding [80,81,82,83]. Independent physicochemical studies converge on charge neutralization as the primary driver of chitosan–DNA condensation. Increasing the chitosan/DNA charge ratio promotes the transition of DNA from extended or loosely associated conformations toward compact morphologies, whereas reduced chitosan protonation favors decondensation [84]. Polymer molecular weight further alters particle size and surface charge under optimized formulation conditions [85]. Thus, particle size and morphology are formulation-dependent outputs rather than fixed properties of chitosan. As a formulation example, Liu et al. [31] constructed pH-responsive chitosan–polydeoxyribonucleotide nanoparticles (CP-NPs) through electrostatic self-assembly (Figure 2A). Under mildly acidic preparation conditions, the primary amino groups of chitosan are protonated to form –NH_3_^+^ groups, which associate with the negatively charged phosphate groups of PDRN and drive macromolecular chain collapse into spherical nanoparticles. Adjustment of the amine/phosphate ratio generated nanoparticles of approximately 161 nm and protected PDRN against DNase I degradation [31]. This example links charge stoichiometry to both particle formation and nuclease protection. More generally, nuclease resistance is governed by nucleic acid packing and polymer-mediated shielding rather than particle diameter alone [83]. The pH responsiveness of CP-NPs originates from the reversible protonation of chitosan. At mildly acidic pH relevant to the skin surface, protonated chitosan remains hydrated and positively charged, promoting nanoparticle swelling, local retention, and PDRN release. At near-neutral or slightly alkaline pH, deprotonation decreases the positive charge density of chitosan, weakens chitosan–PDRN association, and induces particle enlargement and structural rearrangement. However, nucleic acid protection and release should be considered coupled formulation variables. For gene-active nucleic acids, chitosan–pDNA studies indicate that intermediate polyplex stability is preferable to either premature dissociation or persistent complexation, because efficient intracellular activity requires polyplex unpacking to occur in temporal coordination with endo/lysosomal escape [86]. Accordingly, no universal N/P ratio defines optimal chitosan-mediated condensation; the appropriate formulation window should instead be established by jointly evaluating nuclease resistance, decondensation or release kinetics, and functional nucleic acid activity.

For cutaneous formulations, however, intracellular gene expression is not necessarily the primary performance endpoint. Chitosan architecture influences the skin-facing biological interface through particle size, surface charge, polymer chain characteristics, and formulation composition, thereby modulating cutaneous adhesion, penetration depth, and local tissue retention. Experimental skin models have shown that chitosan nanoparticles can access viable epidermal and dermal compartments, although the extent of penetration remains strongly dependent on formulation attributes and skin-barrier integrity [87,88]. These observations should not be equated with productive cytosolic delivery. Mechanistic studies of chitosan–DNA polyplexes indicate that the dominant endocytic pathway varies with polymer architecture, whereas direct evidence of endo/lysosomal escape has been obtained primarily in gene-delivery models rather than in topical PDRN systems [86,89]. Accordingly, for skin-directed chitosan–nucleic acid formulations, the more relevant design endpoints are controlled cutaneous deposition, local retention, nuclease protection, and cargo release, whereas transfection or gene-expression readouts should be regarded as application-specific rather than universal measures of delivery performance.

#### 3.1.2. Polyethyleneimine (PEI)

Polyethyleneimine (PEI) provides a representative model for relating polycation architecture to nucleic acid condensation. PEI molecular weight and branching, together with the N/P ratio and ionic environment, determine polyplex compaction and stability [90]. Increasing effective cationic charge generally enhances DNA condensation and protection, whereas overly persistent association can impair intracellular unpacking and increase cytotoxicity. Thus, reversible rather than maximal complexation represents a more relevant design objective for PEI-mediated delivery [91,92]. Kou et al. [93], using AFM-based single-molecule force spectroscopy, showed that PEI binds double-stranded DNA predominantly through electrostatic interactions with the phosphodiester backbone, rather than through groove binding or intercalation. Increasing the N/P ratio enhanced DNA compaction, whereas the interaction strength remained relatively stable between pH 5.0 and 7.4 but decreased with increasing ionic strength (Figure 2B). These findings identify charge stoichiometry and electrostatic screening as major determinants of PEI–DNA association and support formulation-specific optimization rather than a universal N/P ratio [94]. Beyond condensation, PEI-mediated delivery remains strongly formulation-dependent. Endocytic uptake is broadly observed, but the dominant pathway varies with polyplex properties and cell type. The classical proton-sponge hypothesis remains a proposed, non-exclusive explanation for PEI-mediated endosomal escape; membrane destabilization and polymer swelling may also contribute, and buffering capacity alone does not constitute direct evidence of escape. PEI molecular weight, branching, and N/P ratio further influence intracellular trafficking, transgene expression, and tissue distribution, making these outcomes formulation-dependent.

#### 3.1.3. 4-Dimethylaminopyridine (DMAP)

It is critical for the field to establish innovative preparation techniques that are simple, efficient, and environmentally sustainable. Daheui Choi et al. [38] reported the fabrication of nano-sized DNA hollow spheres through an innovative self-templating strategy, using enzymatic DNA microsponges as the core template material (Figure 2C). Electrostatic binding between the nucleophile DMAP and DNA enables the progressive loosening of the entangled structure within microsponges, achieved through a straightforward processing step. Through this procedure, DNA subsequently reassembles into hollow shell-like formations, thus bypassing the conventional requirement for temperature-controlled denaturation and renaturation steps. Notably, this synthetic protocol is conducted exclusively in an aqueous system, eliminating the requirement for organic solvents and thereby yielding biologically compatible and environmentally sustainable products. This methodological breakthrough not only addresses previous limitations in DNA nanostructure fabrication but also opens new avenues for their biomedical implementation.

#### 3.1.4. Polyamine-Mediated Electrostatic Compaction of Nucleic Acids

Natural polyamines, particularly spermidine and spermine, are extensively protonated under physiological conditions and therefore act as multivalent counterions toward the negatively charged phosphate backbone of nucleic acids [95]. Deng et al. [96] systematically investigated the interactions of spermidine and spermine with genomic B-DNAs spanning a broad range of GC contents using Raman difference spectroscopy. Polyamine addition predominantly perturbed the phosphate-associated bands near 785 and 1090 cm^−1^, whereas changes in nucleobase-sensitive markers were substantially weaker, demonstrating that the phosphate backbone constitutes the principal binding interface. On this basis, the authors proposed a largely sequence-independent mechanism in which dynamically distributed polyamines are electrostatically confined near the DNA surface, thereby screening phosphate–phosphate repulsion, facilitating interhelical approach, and inducing condensation without appreciably disrupting base pairing, base stacking, or the native B-form conformation. Thomas et al. [33] subsequently integrated thermodynamic, spectroscopic, and microscopic evidence to examine how this electrostatic interaction governs higher-order DNA organization and its application in gene delivery. Their analysis indicated that progressive neutralization of the DNA linear charge density by multivalent polyamines drives a cooperative coil-to-globule transition, producing toroidal, spheroidal, or rod-like condensates, frequently with dimensions relevant to cellular internalization. The extent and morphology of compaction are controlled by polyamine valency and molecular architecture, the polyamine-to-phosphate ratio, ionic strength, and competition from monovalent counterions. Collectively, these studies establish charge neutralization and counterion-mediated electrostatic correlation as the central mechanism underlying polyamine-induced nucleic acid compaction, while also highlighting a critical design requirement for nonviral delivery systems: electrostatic association must be sufficiently strong to condense and protect nucleic acids, yet sufficiently reversible to permit intracellular decondensation and cargo release.

### 3.2. Metal–Ligand Coordination

Metal ions regulate nucleic acid conformation and higher-order assembly through interactions ranging from nonspecific electrostatic screening to site-specific metal–ligand coordination [97]. Depending on the identity and hydration state of the metal ion, phosphate oxygens and nitrogen- or oxygen-containing groups of the nucleobases can serve as potential binding sites, thereby modulating backbone repulsion, interstrand association, and structural organization [97,98]. Coordination-active metal ions, such as Fe^2+^ and Cu^2+^, can promote nucleic acid condensation or crosslinking through direct interactions with phosphate and nucleobase ligands, whereas Mg^2+^ predominantly stabilizes nucleic acid structures through electrostatic screening and hydrated, outer-sphere interactions. In contrast, Co (NH_3_) _6_^3+^ primarily acts as a multivalent counterion rather than through direct coordination with nucleic acid ligands. These mechanistically distinct interactions provide tunable control over the morphology, stability, and responsiveness of nucleic acid assemblies, thereby offering a physicochemical basis for the rational design of metal-mediated nanostructures for biosensing, bioimaging, and therapeutic delivery [34]. However, the translational value of metal-mediated assemblies cannot be inferred from structural control alone. Oxidation-state stability, metal/ligand exchange in biological fluids, redox reactivity, competitive protein binding, systemic disposition, clearance, and dose-dependent toxicity can alter both assembly integrity and biological exposure and should therefore be evaluated together with assembly chemistry.

#### 3.2.1. Fe^2+^-Mediated Coordination Crosslinking

**Figure 3:**
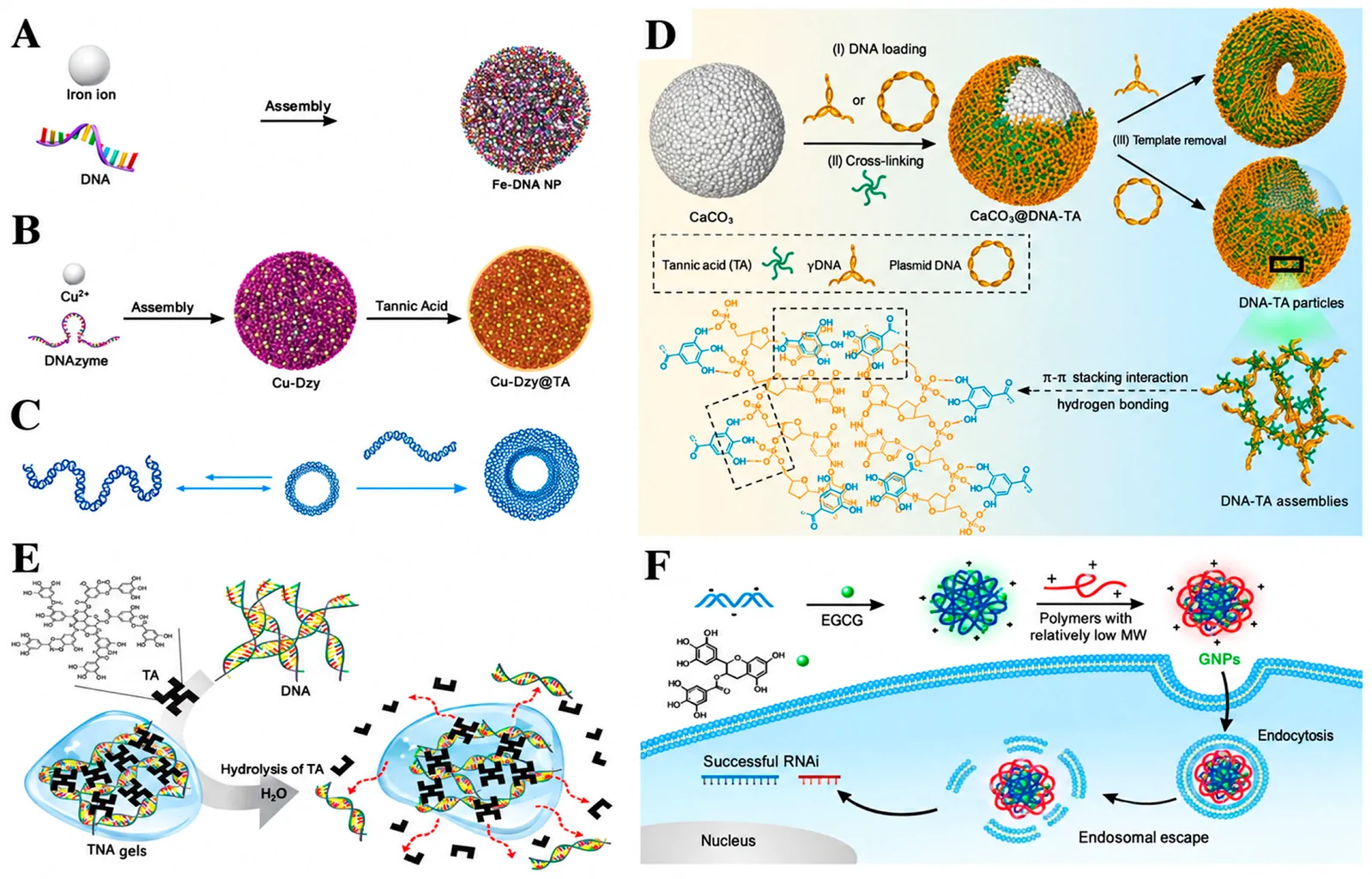
Representative metal-, counterion-, and polyphenol-mediated nucleic acid co-assemblies, in which nucleic acids function as cooperative structural components together with external molecular or ionic partners. (**A**) Schematic depiction of the mechanism for Fe–DNA nanosphere assembly; Reprinted with permission from ref. [34], Copyright (2018) *Angewandte Chemie International Edition*. (**B**) Schematic illustration of Cu-Dzy–TA interactions.; Reprinted with permission from ref. [36], Copyright (2021) *Angewandte Chemie*. (**C**) Schematic Illustration of DNA Condensation by Hexaamminecobalt (III). (**D**) DNA-TA microparticles. Reprinted with permission from ref. [108], Copyright (2023) *Angewandte Chemie International Edition*. (**E**) Schematic description of the formation and degradation of TNA gels. Reprinted with permission from ref. [37], Copyright (2015) *Advanced Functional Materials*. (**F**) Schematic of GNP synthesis and the hypothetical model of gene expression inhibition. Reprinted with permission from ref. [110], Copyright (2018) *ACS Central Science*.

Fe^2+^-mediated coordination provides a direct route from metal–DNA recognition to Fe–DNA nanosphere assembly (Figure 3A) [34]. The Fe^2+^/nucleotide ratio and reactant concentration tune particle dimensions, whereas base-dependent Fe^2+^ affinity (A > G > C > T) modulates molecular packing and DNA loading. The independent formation of Fe–DNA nanospheres by Fe(II)-driven self-assembly further supports the reproducibility of this coordination mechanism [35]. These findings identify coordination stoichiometry and base chemistry as generalizable structural determinants, but do not establish the coordination window that optimally balances nucleic acid protection and release. Moreover, Fe^2+^ is redox-active; oxidation, competitive metal/ligand exchange, and protein binding in biological fluids may alter metal speciation and assembly integrity. Therefore, biodistribution, clearance, redox-related off-target effects, and dose-dependent toxicity should be evaluated directly rather than inferred from particle morphology or colloidal stability [99]. From a translational perspective, Fe^2+^-based assemblies are sensitive to oxidation-state changes and metal exchange in biological media. Because labile iron can promote redox stress and systemic iron is tightly regulated by transferrin- and ferritin-centered homeostasis, serum stability, redox behavior, organ disposition, and dose-dependent toxicity should be evaluated rather than inferred from particle morphology alone.

#### 3.2.2. Cu^2+^–DNAzyme Catalytic Nanohybrids

Cu^2+^–DNAzyme nanohybrids represent a coordination-coupled architecture in which structural assembly and catalytic function are partly separable. Cu^2+^ binds phosphate and nucleobase donor sites, condensing DNAzyme into carrier-free nanospheres that are further stabilized by tannic-acid complexation (Figure 3B) [36]. Similar spherical particles formed from random DNA indicate that gross morphology is governed mainly by metal–nucleic acid coordination, whereas the 10–23 sequence defines RNA-cleavage activity. After intracellular release, Cu^2+^ is reduced by glutathione to Cu^+^ for Fenton-like ROS generation, while the DNAzyme mediates Mg^2+^-dependent VEGFR2 mRNA cleavage. However, quantitative relationships among Cu^2+^/DNAzyme stoichiometry, particle size, nuclease shielding, and intracellular disassembly remain unresolved. For translation, Cu speciation, ligand exchange with biomolecules, and plasma-protein association should be evaluated because they may alter coordination stability and biological fate [100,101]. Fluorescence-based tumor accumulation does not resolve Cu-specific organ retention or excretion, and the reported short-term tolerability does not establish dose-dependent or cumulative metal toxicity. Intracellular trafficking and endosomal release also require direct verification [25]. However, Cu^2+^/Cu^+^ interconversion that supports catalytic activity may also generate off-target redox stress. Because copper is tightly regulated by plasma proteins and dedicated cellular trafficking pathways, Cu–DNAzyme systems require the explicit assessment of protein-induced metal redistribution, systemic disposition, clearance, and dose-dependent toxicity in addition to catalytic efficacy.

#### 3.2.3. Mg^2+^-Regulated Nucleic Acid Folding and Condensation

Mg^2+^ acts primarily as a pathway modifier rather than the principal condensing species in Co (NH_3_) _6_^3+^-driven DNA toroid formation. Mg^2+^ preassociation suppresses toroid nucleation while permitting greater growth of the condensates that form, producing fewer but larger toroids (Figure 3C) [102] This effect depends on ion-addition sequence, total cation concentration, temperature, and dilution, indicating that morphology reflects both nucleation/growth kinetics and charge-compensation thermodynamics. Unlike redox-active transition metals, Mg^2+^ is physiologically regulated and redox-inert; nevertheless, dilution and competition with endogenous ions and biomolecules may alter counterion association and structural persistence. Because systemic Mg^2+^ balance is strongly governed by renal handling, exogenous dose and clearance should also be considered. Thus, the current evidence defines a physicochemical condensation mechanism, whereas the in vivo stability and pharmacokinetics of these assemblies remain insufficiently established.

Unlike Fe^2+^ or Cu^2+^, Mg^2+^ is redox-inert and physiologically abundant; its translational constraints therefore arise mainly from ion exchange, physiological dilution, and systemic ionic load rather than redox toxicity. Because Mg^2+^ balance is strongly controlled by renal excretion, assembly stability and dose should be evaluated under physiological ionic conditions, particularly when clearance is impaired.

#### 3.2.4. Co (NH_3_) _6_^3+^-Induced DNA Toroidal Condensation

Circular DNA (e.g., plasmids and cDNA) serves as the fundamental structural basis for the high-density compression of genomic information and precise functional regulation within living organisms, owing to its unique topological structure and dynamic conformational plasticity. Meanwhile, artificially induced DNA toroids act as biomimetic models of natural DNA compaction, providing crucial insights for elucidating the physicochemical mechanisms underlying DNA aggregation and optimizing gene delivery systems [103]. Co (NH_3_) _6_^3+^ represents a distinct mode of metal-associated nucleic acid assembly in which the intact, substitution-inert Co (III) complex functions primarily as a trivalent condensing counterion. Charge compensation, counterion correlations, and hydration-mediated contacts promote cooperative interhelical association and DNA collapse into toroidal condensates (Figure 3C), without requiring inner-sphere ligand exchange [104]. Toroid dimensions and the toroid/rod balance depend on ion concentration, ionic strength, DNA length and topology, and the competition between nucleation and post-nucleation growth; cryo-EM further reveals densely packed DNA with local hexagonal order. However, links between packing density and nuclease protection or release, productive endosomal escape, and architecture-dependent biodistribution remain insufficiently established. Moreover, coordination inertness should not be equated with pharmacological inertness: redox stability in biological media, systemic distribution, clearance, and dose-dependent toxicity of CoHex–DNA toroids remain poorly defined. Acute studies of unassembled CoHex in mice reported dose-dependent cobalt accumulation, particularly in kidney and liver, but these findings cannot be directly extrapolated to DNA condensates. Thus, CoHex toroids are best regarded as mechanistic models of multivalent-ion-driven DNA condensation until assembly-specific pharmacokinetic and safety relationships are established [105].

### 3.3. Hydrogen Bonding, Base Stacking, and Polyphenol Adhesion

Polyphenol-assisted nucleic acid assembly is primarily governed by multivalent hydrogen-bonding and hydrophobic/aromatic interactions between phenolic groups and nucleobases or phosphate-rich nucleic acid surfaces. Architecture is therefore determined by phenolic-group density, nucleic acid topology, component stoichiometry, and, when present, secondary polymers or templates. These parameters regulate nucleic acid accessibility and thereby couple morphology with enzymatic protection and subsequent cargo release. Current evidence is strongest for TA- and EGCG-assisted systems, whereas curcumin mainly provides molecular-level evidence of nucleic acid association rather than a validated delivery architecture [106,107,108].

#### 3.3.1. Tannic Acid (TA)

Tannic acid (TA) functions as a multivalent supramolecular crosslinker rather than simply a bioactive additive. In DNA–TA assemblies, nucleic acid geometry and template configuration strongly influence the formation of microparticles, capsules, or adhesive networks, whereas dense TA association restricts nuclease accessibility (Figure 3D,E) [37,106]. The reversible nature of these interactions permits structural degradation and cargo exposure; however, a quantitative TA/DNA window that optimally balances protection and release has not yet been established. Although TA-based particles support cellular delivery and in vivo immune activity, direct evidence for endosomal escape and architecture-dependent biodistribution remains limited, and independent validation of identical TA–DNA architectures is still scarce.

#### 3.3.2. Curcumin

I Curcumin is better interpreted as a molecular-interaction model than as an established nucleic acid carrier [109,110]. Spectroscopic evidence indicates binding to DNA grooves, nucleobases, and phosphate groups, accompanied by concentration-dependent aggregation without major disruption of canonical DNA or RNA conformations [111]. These observations support noncovalent polyphenol–nucleic acid association but do not establish controlled nanoscale condensation, nuclease protection, endosomal escape, or in vivo disposition. Curcumin should therefore be used here to define the interaction mechanism rather than to imply a validated delivery platform [112].

#### 3.3.3. Epigallocatechin-3-O-gallate

EGCG provides stronger structure–function evidence because it can precomplex siRNA through cooperative hydrogen-bonding and hydrophobic interactions and subsequently recruit cationic polymers to form core–shell nanoparticles (Figure 3F) [108]. Particle formation depends on the EGCG/siRNA and polymer/siRNA ratios and on polymer architecture, while EGCG-mediated preorganization enables low-molecular-weight polymers to condense and protect siRNA with reduced cationic burden. Importantly, enhanced uptake is not mechanistically universal: EGCG–siRNA/polymer nanoparticles exhibit lipid-raft-associated internalization, whereas HA–EGCG ternary complexes undergo CD44-mediated endocytosis. Reduced endolysosomal colocalization, cytosolic siRNA release, and functional gene silencing support productive intracellular delivery in the former system, although direct quantification of membrane escape remains limited. Thus, enhanced nucleic acid stabilization is observed across independent EGCG-containing systems, whereas uptake route and subsequent intracellular fate remain architecture-dependent [107,113,114].

### 3.4. Surfactant- and Membrane-Assisted Co-Assemblies

**Figure 4:**
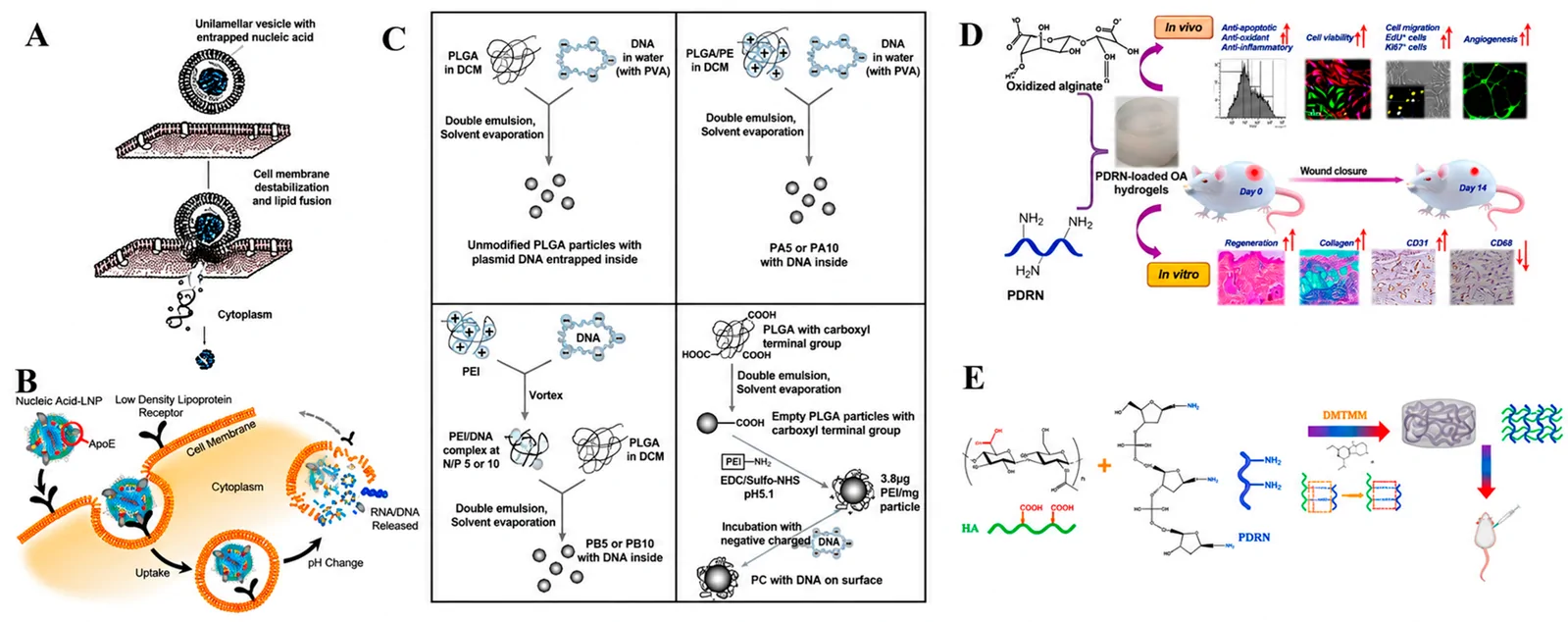
Representative transition from membrane-assisted nucleic acid co-assembly to carrier- and matrix-dictated nucleic acid systems. In the latter systems, the carrier or matrix predominantly defines the overall architecture, whereas nucleic acids mainly provide genetic, therapeutic, or regenerative functions. (**A**) Proposed mechanism of Mg^2+^-induced cellular internalization of neutral liposome–DNA formulations. Reprinted with permission from ref. [120], Copyright (2005) *Il Farmaco*. (**B**) Self-assembly of nucleic acid-loaded lipid nanoparticles. Reprinted with permission from ref. [122], Copyright (2018) *Multiple Myeloma*. (**C**) DNA microsponges. Reprinted with permission from ref. [128], Copyright (2024) *International Journal of Biological Macromolecules*. (**D**) Schematic representation of preparation, and characterization of PDRN-loaded OA hydrogel and its in vitro and in vivo studies. Reprinted with permission from ref. [132], Copyright (2024) *International Journal of Biological Macromolecules*. (**E**) The fabrication procedure of HP cross-linked hydrogel. Reprinted with permission from ref. [41], Copyright (2024) *International Journal of Biological Macromolecules*.

Cationic surfactants have demonstrated notable efficacy in facilitating the transport and condensation of DNA [115]. The adsorption of cationic liposomes onto DNA backbones diminishes the electrostatic repulsion between neighboring phosphate groups, enabling their closer proximity and resulting in the phenomenon referred to as DNA compaction [116,117]. Erhan prepared different complex systems containing calf thymus DNA,1,2-dipalmitoyl-sn-glycero-3-phosphorylcholine (DPPC) liposomes, and Mg^2+^ or C12 TMA (N-alkyl- N, N, N-trimethylammonium ion, (*n* = 12)) ternary complexes [118]. Nucleic acid compaction capacities of Mg^2+^ trimetylammonium ions (Cn and N-alkyl-N, N, NTMA, *n* = 12) were compared, with regard to surface interaction with unilamellar vesicles. Differential scanning microcalorimetry was employed to characterize synthetic phosphatidylcholine liposomes, calf thymus DNA, and their ternary complexes formed with magnesium ions and dodecyltrimethylammonium (Figure 4A). An initial compaction of DNA using detergent, followed by the subsequent introduction of liposomes, yields uniform and colloidally stable nanocomplexes with minimal size dispersion. Michela Pisani et al.’s DPPC-DNA-Me^2+^ ternary complexes were prepared using metal cations as a bridge connecting the polar head of negatively charged DPPC and the phosphate group of DNA [119]. The thermotropic phase behavior of bound-state lipids in the complexes was partially altered compared to pure DPPC. The methodology represents a viable platform for advancing gene-based therapies. Investigating complex architecture and phase behavior provides mechanistic insights into lipid–DNA recognition processes, guiding the design of optimized synthetic gene delivery systems.

**Table 3:** Mechanism-centered comparison of supramolecular nucleic acid co-assemblies.

Dominant Assembly Mechanism	Representative Partners	Principal Nucleic Acid Binding Site(s)	Typical Structural Outcome	Key Control Variables	Structure–Function Consequence	Major Design Trade-Off
Polycation-mediated electrostatic complexation [31,38,93]	Chitosan, PEI and DMAP	Anionic phosphate backbone; additional hydrogen bonding may contribute	Compact polyplexes, spherical nanoparticles, or polymer-confined nucleic acid domains	Polymer molecular weight and architecture, N/P ratio, degree of protonation, ionic strength, nucleic acid length, and mixing history.	Charge neutralization controls polyplex compaction and nuclease protection, whereas polymer architecture and complex stability influence cellular uptake and intracellular unpacking.	Increasing charge density favors condensation and protection but can increase cytotoxicity and suppress cargo release; buffering capacity alone does not establish endosomal escape.
Polyamine-induced cooperative compaction [33,96]	Spermidine, spermine, and other multivalent amines	Phosphate backbone through multivalent electrostatic neutralization	Globular or toroidal DNA condensates with biomimetic packing density	Polyamine valency and concentration, DNA length, ionic composition, and temperature	High-density packaging and reduced molecular dimensions without covalent modification	Condensate stability and reversibility are highly sensitive to ionic conditions
Specific metal–ligand coordination [34,35]	Fe^2+^ and other coordinatively active metal ions	Phosphate oxygens and nucleobase nitrogen/oxygen donor atoms	Coordination-crosslinked nanospheres or networked assemblies	Metal-to-nucleotide ratio, reactant concentration, nucleobase composition, pH, and metal oxidation state	Morphological control, high nucleic acid loading, and integration of metal-derived catalytic or imaging functions	Coordination improves stability but introduces metal-dependent toxicity and redox-control requirements
Coordination-coupled catalytic co-assembly [36]	Cu^2+^, DNAzyme, and tannic acid	Phosphate/base donor atoms and phenolic coordination sites	Carrier-free Cu–DNAzyme nanohybrids with a stabilizing metal–polyphenol layer	Cu^2+^/DNAzyme ratio, sequence, incubation conditions, tannic-acid coating, pH, and reducing environment	Co-localization of gene catalysis and copper-mediated catalytic activity within one nanosystem	Catalytic amplification must be balanced against off-target redox stress and metal clearance
Counterion-mediated condensation [103,104]	Co(NH_3_)_6_^3+^ with Mg^2+^ as a kinetic and thermodynamic modulator	Predominantly nonspecific association with the phosphate backbone	DNA toroids with ion-dependent nucleation, dimensions, and packing order	Ion valency, ion addition sequence, pre-binding, DNA concentration, and ionic strength	Sequence-independent control over DNA collapse and toroid organization	Preparation-history dependence and insufficiently defined assembly-specific pharmacokinetics and metal-related safety.
Polyphenol-mediated multivalent association [106,114]	Tannic acid and EGCG	Phosphate groups, nucleobases, and polymer-associated hydrogen-bonding/electrostatic sites	Microparticles, capsules, adhesive gels, or ternary RNA nanocomplexes	Polyphenol/nucleic acid ratio, pH, oxidation state, polymer charge density, and component-addition sequence	Improved serum stability, tissue adhesion, co-delivery, cellular internalization, and immunostimulatory presentation	Interaction heterogeneity and oxidation-dependent behavior complicate mechanistic quantification
Surfactant- or membrane-assisted condensation [116]	Cationic surfactants	Phosphate backbone and lipid headgroup interface	Lipoplexes, multilamellar structures, or DNA–lipid–metal ternary phases	Surfactant structure, lipid phase, charge ratio, cation identity, mixing sequence, and temperature	Coupled DNA compaction and membrane association can enhance colloidal stability and cellular contact	Phase heterogeneity, lipid fusion, and charge-dependent toxicity limit reproducibility

N/P ratio denotes the molar ratio of protonatable nitrogen to nucleic acid phosphate. The table separates specific metal coordination from nonspecific counterion condensation.

### 3.5. Nucleic Acid-Loaded Composite Biomaterials

Within this class, formulation criteria are cargo-dependent: sequence-defined nucleic acids require preservation and delivery of sequence-encoded activity, whereas PDRN-containing systems are governed primarily by matrix-controlled retention, release, and preservation of local bioactivity. Table 4 provides a synopsis of composite biomaterials incorporating nucleic acids.

#### 3.5.1. Lipid Nanoparticles

Lipid nanoparticles (LNPs) represent well-established platforms in biopharmaceutical applications, enabling effective nucleic acid encapsulation and intracellular transport [120]. LNP benefits extend to fundamental research applications, where commercially accessible microfluidic production enables preparation at scales suitable for in vitro cellular studies [39]. Within the present role-based framework, LNPs are carrier-dominated assemblies whose architecture is set mainly by lipid chemistry and processing. During rapid acidic mixing (Figure 4B), protonated ionizable lipids electrostatically complex nucleic acids and drive encapsulation; after neutralization, the apparent pKa is a key determinant of ionization behavior under physiological and endosomal conditions [39]. Helper phospholipids regulate lipid packing and membrane fusogenicity and cholesterol tunes internal packing and structural stability, whereas PEG-lipids suppress aggregation and constrain particle size, although excessive or persistent PEG can hinder cellular interactions [121]. Together with lipid/cargo stoichiometry and mixing kinetics, these variables determine particle size, morphology, internal organization, and encapsulation efficiency [122]. In biological fluids, adsorption of plasma proteins forms a dynamic protein corona that modifies cellular recognition and biodistribution, making organ tropism formulation-dependent [123]. Endosomal acidification can promote ionizable-lipid-mediated membrane destabilization, but internalization should not be equated with productive escape [124]. PEGylation also introduces a translational trade-off because anti-PEG antibodies may alter clearance and immune responses [124]. Finally, aggregation, lipid/RNA degradation, and process variability constrain formulation stability and scale-up; manufacturing should therefore preserve critical quality attributes, including particle-size distribution, encapsulation, and molecular integrity. Recent LNP development has shifted from generic nucleic acid encapsulation toward organ- and cell-selective RNA delivery through ionizable-lipid chemistry and formulation-dependent biological interactions. Importantly, endosomal escape is now recognized as both a limiting step for productive cytosolic delivery and a potential source of inflammatory membrane damage, linking delivery efficiency, biodegradability, tissue targeting, and innate immune activation within the same formulation design space [125].

#### 3.5.2. Polymeric Carrier-Confined Systems

Polymeric carrier-confined systems introduce hierarchical structural control by embedding nucleic acid polyplexes within a secondary polymer matrix. In these systems, polyplex formation governs molecular-scale cargo condensation, whereas matrix composition and fabrication conditions regulate particle organization, cargo retention, and release behavior. Comparative PLGA/PEI/pDNA formulations prepared using different fabrication strategies (Figure 4C) demonstrate that processing conditions can markedly influence buffering behavior, cellular uptake, transfection efficiency, and cytocompatibility, indicating that the polymer matrix actively modulates delivery performance rather than functioning as a passive container [126,127]. Rational design should therefore balance sufficient nucleic acid protection with timely intracellular decomplexation while minimizing excessive cationic exposure. Importantly, enhanced cellular uptake or transgene expression alone does not demonstrate efficient endosomal escape, which requires direct intracellular trafficking evidence. Because these relationships remain formulation-dependent, broader validation is required before general architecture–performance relationships can be established.

#### 3.5.3. PDRN-Loaded Alginate Hydrogels

In the present framework, PDRN is treated as a heterogeneous DNA-fragment bioactive cargo rather than a sequence-programmed gene therapeutic. Its relevance to composite biomaterials lies primarily in matrix-mediated protection, local retention, and the controlled presentation of regenerative activity [40,128,129]. Alginate–PDRN hydrogels are better interpreted as network-regulated local delivery systems rather than as isolated wound-healing formulations. In oxidized alginate systems, ionic crosslinking and Schiff-base coupling establish the matrix, whereas crosslink density, swelling, and degradation are expected to govern PDRN mobility and tissue exposure (Figure 4D) [130]. Dananjaya and colleagues [130] reported improved wound-repair outcomes in preclinical models. These data support proof-of-concept bioactivity but not clinical efficacy; the relationship between hydrogel network properties, PDRN exposure, and biological response remains unresolved. Independent validation across formulations is still limited. Future studies should therefore correlate matrix parameters with release kinetics and local pharmacological exposure rather than relying primarily on wound-closure outcomes. Independent validation across PDRN hydrogel formulations remains limited.

#### 3.5.4. PDRN-Integrated Hyaluronic Acid Hydrogels

Injectable hyaluronic acid (HA) hydrogels are widely used for soft-tissue augmentation because their viscoelasticity, hydration capacity, and biodegradability enable volume restoration and tissue support [131,132]. In HA–PDRN composite systems, HA primarily forms the structural matrix, whereas PDRN serves as a non-sequence-defined regenerative bioactive component with reported anti-inflammatory and collagen-supporting activities [9]. Guo et al. developed an injectable HA–PDRN crosslinked hydrogel in which the carboxyl groups of HA were covalently coupled to amino groups of PDRN through amide-bond formation (Figure 4E) [41]. The resulting hydrogel exhibited suitable viscoelasticity, injectability, hydration, biodegradability, and biocompatibility. In a murine implantation model, the HA–PDRN hydrogel maintained volume for more than 24 weeks and was associated with attenuated inflammatory responses and enhanced collagen deposition. These findings indicate that covalent incorporation of PDRN can integrate the viscoelastic and hydrating functions of the HA matrix with the regenerative activity of PDRN. However, the available evidence remains preclinical, and further studies are required to determine how formulation and crosslinking parameters affect injectability, degradation kinetics, long-term biocompatibility, and reproducibility before clinical translation.

**Table 4:** Nucleic Acid-Loaded Composite Biomaterials.

Platform	Architecture-Forming Phase	Nucleic Acid/Bioactive Component	Incorporation Strategy	Function of the Matrix	Function of the Nucleic Acid Component	Dominant Application Value	Principal Limitation
Lipid nanoparticles [39,133]	Ionizable lipid/helper lipid/cholesterol/PEG-lipid phase	siRNA, mRNA, or pDNA	Rapid mixing and encapsulation during lipid self-assembly	Protects cargo, controls particle size and biodistribution, and supports endosomal release	Provides sequence-specific therapeutic information	Systemic gene therapy, vaccination, and genome editing	Organ tropism, immune activation, storage stability, and scale-up
Polymeric carrier-confined systems [127]	PLGA or PLA-mPEG matrix containing nucleic acid polyplex domains	pDNA or PEI-DNA polyplexes	Double emulsion, solvent evaporation, or related matrix-solidification methods	Provides protection, depot residence, aerosol properties, and sustained release	Supports gene expression after release and unpacking	Pulmonary or local gene delivery	Processing damage, burst release, incomplete liberation, and cationic toxicity
Alginate-PDRN hydrogels [130]	Ionically and Schiff-base-crosslinked oxidized alginate network	PDRN	Physical entrapment and local retention within a hydrated polysaccharide matrix	Provides a moist wound environment, structural support, and sustained local presentation	Supplies regenerative and anti-inflammatory signaling activity	Diabetic wound repair and regenerative dressings	Burst release, wet-state mechanics, sterilization, and PDRN batch variability
Hyaluronic acid-PDRN hydrogels [41]	Crosslinked hyaluronic acid network	PDRN	Integration within an injectable soft-tissue matrix	Provides viscoelastic filling, hydration, tissue support, and controlled residence	Contributes regenerative, anti-inflammatory, and collagen-supporting activity	Dermal filling, skin rejuvenation, and soft-tissue repair	Degradation-rate matching, injection performance, long-term safety, and product classification

## 4. Applications of Advanced Nucleic Acid Architectures

### 4.1. Drug Delivery

The field of drug delivery systems represents a pivotal domain within the realms of modern medicine and biotechnology, with the overarching objective being the enhancement of drug targeting, the mitigation of adverse effects, and the augmentation of therapeutic efficacy. In recent years, the advent of sophisticated nucleic acid assembly techniques has precipitated revolutionary breakthroughs in the domain of drug delivery. The team led by Zhan Yurong [134] utilized plasmid DNA as the payload, oxidized glutathione-crosslinked low-molecular-weight polyethyleneimine (GP) as the cationic scaffold, and sodium alginate as the anionic regulatory component, thereby developing structurally stable ternary spherical SA@GP/DNA nanoparticles. In this system, the GP molecular chains are rich in protonated amino groups (—NH_3_^+^), which can bind to the phosphate anions (PO_4_^3−^) on the phosphate backbone of plasmid DNA via electrostatic attraction. This binding results in the compression of the DNA to form a binary GP/DNA complex. Subsequently, the carboxylate anions (—COO^−^) carried by the mannuronic and glucuronic acid units in the sodium alginate molecules interact electrostatically with the free —NH_3_^+^ groups on the GP that are not bound to DNA. Concurrently, the peptide bonds in the GP, hydroxyl groups in sodium alginate form weak hydrophobic interactions, and hydrogen bonds with DNA base pairs, complementing the electrostatic interactions. This study illustrates an important design principle for advanced nucleic acid delivery systems: delivery performance is not determined by electrostatic DNA condensation alone, but by the coordinated optimization of condensation strength, surface charge, nuclease protection, cellular uptake, endosomal escape, intracellular release, and biosafety. Compared with single-component carriers, programmable ternary or multicomponent co-assemblies provide a more flexible strategy for dual-gene or multi-gene delivery, particularly in diseases requiring coordinated regulation of multiple pathways, such as cancer, inflammatory disorders, and regenerative repair. From a translational perspective, these systems may also be integrated with imaging agents, photosensitizers, or upconversion nanomaterials for spatiotemporally controlled therapy; however, such combinations should be presented as future engineering directions unless direct experimental evidence is available [135]. Table 5 provides a synopsis of the primary domains of application for advanced nucleic acid structures.

### 4.2. Intracellular and Local Nucleic Acid Delivery

Delivery performance should be defined by experimentally resolved transport steps rather than by total cellular accumulation. Tissue access and cellular internalization indicate entry into the target compartment, whereas productive intracellular delivery additionally requires release from endosomal/lysosomal trafficking and access by the nucleic acid to its site of action [136]. Quantitative imaging of LNP–siRNA systems has shown that endosomal escape can be highly inefficient, demonstrating that uptake cannot be used as a surrogate for cytosolic availability or functional delivery [137]. The relevant endpoint is therefore cargo- and route-dependent. For locally administered formulations whose intended activity does not require intracellular gene regulation, local exposure, controlled release, and preserved bioactivity may be more relevant than transfection [31,130]. In contrast, siRNA and mRNA require cytosolic access, whereas pDNA additionally requires nuclear entry [45,121]. Accordingly, uptake, penetration, endosomal escape, intracellular delivery, and transfection should be used only when supported by the corresponding experimental evidence [138]. Table 5 provides a synopsis of the primary domains of application for advanced nucleic acid structures.

### 4.3. Biosensing and Molecular Diagnostics

Biosensors are tools that convert biological signals into recordable signals [139]; with the advantages of high specificity, rapid analysis, high accuracy and low cost, they have gained attention as an alternative method for early diagnosis of cancer. Despite considerable progress in nanobiosensor development, a significant limitation persists. The elevated density of many nanostructures induces molecular aggregation at the sensing interface, consequently diminishing biorecognition efficiency [140]. Qiutong Chen et al. [141] developed a carbon dot-assisted DNA nanosphere platform by the room-temperature self-assembly of polyethylenimine-modified carbon dots (PEI-CDs) with DNA, forming nanostructures termed CANs. In this system, the ultrasmall size and cationic surface of PEI-CDs drive electrostatic complexation with the negatively charged phosphate backbone of DNA, enabling dense DNA loading while protecting nucleic acid probes from enzymatic degradation. This design illustrates a typical nucleic acid–nanomaterial co-assembly strategy in which carbon dots provide fluorescent and cationic assembly functions, whereas DNA contributes molecular recognition, programmability, and target-responsive signal generation. Importantly, Chen et al. [141] further incorporated Mg^2+^ into CANs to construct Mg@CANs and designed DNAzyme-based AND/OR logic-gate systems capable of responding to endogenous human apurinic/apyrimidinic endonuclease 1 and distinguishing miRNA-21 and miRNA-155 expression in tumor cells and tumor sites. Thus, CAN-based biosensors do not merely improve probe loading; they provide a programmable nucleic acid architecture that couples probe protection, cellular delivery, logic biocomputing, and miRNA imaging. From a structure–function perspective, this example demonstrates how controlled nucleic acid condensation and nanointerfacial organization can enhance the sensitivity, stability, and biological applicability of miRNA biosensing for early tumor diagnosis.

Beyond fluorescence-based sensing, nanopore technology offers a complementary approach for label-free single-molecule analysis through direct electrical readout. Under an applied transmembrane potential, individual molecules passing through nanoscale pores generate characteristic ionic current fluctuations, in which blockade amplitude, duration, and temporal patterns reflect molecular size, charge distribution, conformation, and transport behavior [142]. Structural DNA nanotechnology has further expanded nanopore sensing by enabling precise control over pore geometry and chemical functionality. DNA origami structures integrated with solid-state nanopores or engineered as membrane-spanning channels have facilitated programmable molecular translocation and single-molecule nucleic acid recognition through sequence-specific functionalization [143,144]. In addition, hydrophobic modification strategies allow DNA-based nanostructures to insert into lipid membranes, generating artificial nanopores with defined architectures and tunable ionic conductance properties [52,53].

The sensing performance of DNA-based nanopores is governed not only by pore dimensions but also by dynamic structural features, including conformational flexibility, ion distribution, membrane interactions, and analyte transport behavior. Voltage-induced deformation of DNA origami nanopores highlights the importance of mechanical adaptability in regulating ionic signals [145]. Accordingly, computational approaches, including all-atom molecular dynamics and coarse-grained simulations, have become valuable tools for elucidating membrane insertion, hydration structure, ion transport, electro-osmotic effects, and conformational transitions, thereby providing mechanistic guidance for rational nanopore design [53,142].

Recent advances have further promoted the transition of nucleic acid-based sensing systems from fundamental transport studies toward clinically relevant diagnostic applications. Programmable DNA nanostructures combined with nanopore platforms have enabled multiplexed single-molecule detection of antibodies [146], while aptamer-functionalized DNA origami carriers have supported the sensitive identification of individual protein biomarkers, including C-reactive protein in complex biological samples [54,147]. Moreover, shape- and size-programmable DNA membrane nanopores have demonstrated direct label-free protein sensing and portable electrical detection capabilities [54]. Dynamically regulated DNA nanopores further provide opportunities to couple molecular recognition with controlled transport and low-noise signal acquisition [148]. Collectively, these developments highlight structural DNA nanotechnology as a powerful interface between programmable nucleic acid engineering and next-generation molecular diagnostics. Nevertheless, challenges including structural heterogeneity, membrane integration efficiency, nuclease stability, signal variability, and manufacturing reproducibility remain critical barriers toward practical clinical translation. Recent biosensing studies increasingly emphasize architecture-controlled probe presentation, intracellular robustness, and multiplexed signal processing rather than proof-of-concept nanostructure formation alone. Framework-nucleic-acid probes and dynamically tunable DNA nanopores now provide complementary routes toward intracellular imaging and label-free single-molecule detection, although matrix effects, device integration, structural reproducibility, and scalable fabrication remain major barriers to clinical implementation [149]. Table 5 provides a synopsis of the primary domains of application for advanced nucleic acid structures.

### 4.4. Skin Repair and Cosmetic Science

In regenerative tissue repair, nucleic acid-derived bioactivity should be interpreted separately from carrier-mediated formulation effects. Evidence should be considered hierarchically: cell and receptor studies establish mechanistic plausibility, animal models define preclinical tissue responses, observational clinical studies provide exploratory human evidence, whereas randomized trials support only indication-specific clinical efficacy. Among them, polynucleotides and PDRN, mostly derived from fish DNA, have been investigated for improving skin texture, hydration, elasticity, wound repair, scar remodeling, and photoaging-related damage [150]. Their biological activity is generally associated with nucleotide supplementation through the salvage pathway and, in the case of PDRN, activation of adenosine A2A receptor-mediated anti-inflammatory and tissue-repair signaling [9]. Accordingly, PDRN-containing dermatological formulations should be interpreted as regenerative biomaterial systems rather than sequence-specific gene therapies [10]. A randomized, double-blind, placebo-controlled trial in chronic diabetic foot ulcers reported improved healing outcomes with PDRN, providing clinical evidence for that indication but not for generalized tissue regeneration [151]. In parallel, DNA–surfactant/PLA nanofibers illustrate how electrostatic complexation enables nucleic acid incorporation into fibrous matrices [152], whereas biological efficacy and safety require independent validation. Thus, clinical translation should determine whether architecture-dependent residence and release produce measurable tissue-repair benefits rather than infer efficacy from preclinical material performance. Table 5 provides a synopsis of the primary domains of application for advanced nucleic acid structures.

### 4.5. Vaccination, Immunomodulation, and Emerging Biointerfaces

Advanced nucleic acid architectures provide programmable platforms for vaccination and immunomodulation by integrating nucleic acid cargo with materials that regulate molecular protection, tissue delivery, and immune activation. Nucleic acid vaccines deliver genetic templates that direct in situ antigen expression and can be rapidly reconfigured by altering the encoded sequence [153]. As a representative example, Li et al. developed a thermostable dissolving microneedle platform for the intradermal delivery of chitosan oligosaccharide (COS)-encapsulated DNA vaccines encoding the SARS-CoV-2 spike and nucleocapsid antigens [154]. At the formulation level, electrostatic association between protonated amino groups of COS and the anionic phosphate backbone of plasmid DNA condenses the nucleic acid into positively charged nanoparticles. Incorporation of these nanoparticles into poly(vinyl alcohol)/sucrose microneedles enables penetration of the stratum corneum, followed by rapid dissolution and local release in the skin. The formulation facilitates cellular uptake and transfection and promotes dendritic-cell maturation and antiviral immunity through cGAS–STING-mediated interferon signaling. In mice, microneedle-mediated intradermal immunization generated neutralizing antibody responses comparable to intramuscular vaccination while eliciting enhanced systemic and mucosal T-cell responses with prolonged persistence. Notably, the microneedle formulation retained immunogenicity after storage at room temperature for at least one month, highlighting the potential of material-assisted nucleic acid delivery to improve vaccine stability and administration. Further translation will require systematic evaluation of dose uniformity, manufacturing reproducibility, skin tolerability, long-term storage stability, and the balance between productive innate immune activation and nonspecific inflammation. Recent studies further demonstrate that nucleic acid architecture can directly regulate immune signaling. Nanometer-scale control of CpG spacing on DNA origami modulates dendritic-cell activation and downstream adaptive immunity, whereas mRNA–LNP immunogenicity reflects coupled contributions from RNA sensing, lipid composition, and endosomal membrane damage. Thus, productive adjuvanticity and nonspecific inflammatory toxicity should be considered formulation-dependent and mechanistically coupled outcomes [155]. Table 5 provides a synopsis of the primary domains of application for advanced nucleic acid structures.

**Table 5:** Application-oriented platform selection and translational priorities.

Application Domain	Dominant Performance Requirement	Preferred Architecture(s)	Architecture-Derived Advantage	Key Efficacy Endpoint	Major Translational Bottleneck	Priority Development Criterion	Evidence Level	Principal Safety Considerations
Small-molecule and combination drug delivery [15,29,30,50,62]	High loading, spatial control, targeting, protection, and controlled release	DNA origami, tFNAs, SNAs, DNA hydrogels, and polymeric composite particles	Programmable cargo placement or prolonged local retention with modular surface functionalization	Target-site accumulation, intracellular drug availability, therapeutic index, and release–response correlation	Scale-up, release reproducibility, and cross-platform dose normalization	Link critical structural attributes to delivered dose and therapeutic benefit.	Predominantly in vitro/in vivo; clinical evidence remains limited for the listed architectures.	Off-target exposure, carrier persistence, immunogenicity, and degradation-product toxicity.
Gene therapy and gene regulation [39,47,93,127]	Nuclease protection, cellular uptake, endosomal escape, unpacking, and sustained expression/silencing	LNPs, PEI/chitosan polyplexes, PLGA/PEI microparticles, SNAs	Condensation and carrier confinement improve stability, while ionizable/protonatable components promote intracellular release	Expression/silencing efficiency, duration, and cell specificity.	Endosomal escape, extrahepatic targeting, and formulation-dependent biodistribution	Co-optimize protection, escape, unpacking, and functional delivery	Clinical for selected LNP products; phase 0 human evidence for SNA; otherwise mainly preclinical/in vivo.	Innate immune activation, cationic/ionizable-material toxicity, off-target gene effects, and repeat-dose tolerability.
Biosensing and molecular diagnostics [29,50,141]	Probe accessibility, low background, multivalent capture, signal amplification, and logical discrimination	tFNA interfaces, molecular beacons, DNA origami devices, and programmable DNA nanospheres	Defined geometry improves probe orientation and supports multistep or logic-gated molecular recognition	Limit of detection, selectivity, response time, matrix tolerance, and reproducibility	Biofouling, calibration complexity, device integration, and storage stability	Validate performance in clinically relevant matrices rather than buffer-only systems	Predominantly in vitro/ex vivo; limited in vivo/clinical validation.	For in vivo use: biocompatibility, immune response, and material clearance.
Wound healing, skin repair, and aesthetic medicine [29,31,41,130]	Local retention, moisture balance, biocompatibility, inflammation control, and regenerative signaling	Alginate–PDRN and HA–PDRN hydrogels, chitosan–PDRN nanoparticles, and tFNA-based systems	Matrix-mediated residence combines with nucleic acid-derived regenerative or anti-inflammatory activity	Wound closure, collagen organization, angiogenesis, inflammatory markers, tissue mechanics, and durability	PDRN standardization, dose definition, formulation reproducibility, and product classification	Define composition–dose–response relationships against clinically relevant comparators	Clinical evidence exists for PDRN/PN therapy; formulated PDRN and tFNA systems remain mainly preclinical/in vivo.	Sterility, local inflammation/hypersensitivity, degradation products, and chronic/repeat-dose safety.
Vaccination and immunomodulation [31,37,39,48,106]	Co-delivery, antigen-presenting-cell uptake, controlled innate stimulation, and durable adaptive immunity	DNA–TA microparticles, chitosan–DNA nanoparticles, LNPs, and SNAs	Multivalent organization enables co-localization of nucleic acid adjuvants, antigens, and targeting motifs	Antigen-specific cellular/humoral responses, mucosal immunity, and immune durability	Formulation stability, cold-chain dependence, dose optimization, and platform-specific targeting	Define an immune-activation window that preserves potency without excessive inflammation	Clinical for LNP–mRNA vaccines; other listed architectures remain mainly preclinical/in vivo.	Reactogenicity, excessive innate inflammation, anti-carrier responses, and repeat-dose tolerability.

## 5. Translational Considerations and Readiness

Translation requires that an experimentally defined nucleic acid architecture can be expressed as a measurable and reproducible product specification. For sequence-programmed designs, quality control begins with oligonucleotide synthesis and purification, followed by confirmation of sequence identity, strand stoichiometry, and the proportion of correctly assembled structures [25,51]. For supramolecular co-assemblies, the analytical focus shifts toward assembly-state distributions, component ratios, and residual unbound partners, whereas carrier-dominated formulations additionally require verification that cargo integrity and functional release are maintained [34,98]. Because a single size, imaging, or morphology measurement cannot adequately define these complex products, orthogonal physicochemical and functional assays are required. Lot-release criteria should remain within predefined limits after process scale-up and transfer to GMP-compatible manufacture, together with stability-indicating testing under the intended storage conditions, an appropriate sterilization or aseptic strategy, and defined limits for endotoxin and process-related impurities [25,137].

A second requirement is to establish what chemical or structural entity is actually being measured in vivo. Total fluorescence or elemental signal does not necessarily represent an intact nucleic acid architecture after administration. Where technically feasible, pharmacokinetic and biodistribution studies should therefore distinguish the assembled construct from released nucleic acid, carrier-associated species, and relevant degradation products [25,119]. The duration of toxicological evaluation should reflect the expected exposure: repeated administration, slowly cleared components, persistent cationic materials, or inorganic constituents may require longer observation than rapidly degradable formulations. Thus, translational safety studies should be determined by the persistence and administration profile of the final architecture rather than by a uniform testing scheme.

Human evidence remains strongly platform-dependent. LNPs currently provide the most established clinical precedent among the delivery architectures considered here, with the approved siRNA therapeutic patisiran and LNP-formulated mRNA vaccines demonstrating scalable pharmaceutical manufacture and human deployment [122,156]. SNAs have reached early human investigation, exemplified by the phase 0 first-in-human NU-0129 study in recurrent glioblastoma, which evaluated safety, pharmacokinetics, intratumoral accumulation, and target modulation [157]; recent analyses nevertheless indicate that broader SNA translation remains at an early stage [158]. Therapeutic DNA origami systems also remain preclinical despite increasingly sophisticated in vivo validation in animal models [59,60], while tFNA-based therapeutics are similarly supported predominantly by preclinical evidence, with manufacturing, pharmacokinetic, stability, and long-term safety requirements remaining important translational considerations [68,159]. PDRN and PN materials have been evaluated clinically in regenerative and dermatological settings; however, recent systematic assessments continue to identify heterogeneity in formulation, treatment protocols, outcome measures, sample size, and overall evidence quality [R9,R10]. These differences should therefore be interpreted as distinctions in evidence level rather than as intrinsic superiority of one architecture over another [160,161].

## 6. Conclusions

Across the three structural classes discussed in this Review, nucleic acid architecture should be considered a formulation variable that governs molecular protection, transport, intracellular availability, and release rather than an endpoint in itself. Several design principles emerge from the structure–function relationships examined here. First, nucleic acid protection and cargo release should be optimized as coupled variables: stronger condensation can improve nuclease resistance but may also hinder intracellular decomplexation and cargo release [32,33,102,162]. Second, cellular uptake alone should not be used as a surrogate for productive delivery; endosomal escape, intracellular unpacking, cargo availability, and the intended biological response should be evaluated together [102,133,152,154,162]. Third, additional structural complexity should be retained only when it produces a measurable improvement in delivery or pharmacological performance relative to a simpler formulation [25,26,50,143]. Fourth, biosafety should be assessed at the architecture level because carrier composition, surface charge, degradation behavior, and immune activation can influence toxicity independently of nucleic acid sequence [25,102,115,133,154]. Finally, manufacturability should be incorporated during formulation design through the control of composition, particle size, cargo loading, batch reproducibility, storage stability, and scalable processing rather than evaluated only after biological optimization [25,133,135,154].

These principles shift the emphasis from maximizing structural complexity to establishing quantitative structure–performance relationships. For translational development, future studies should link critical structural attributes to delivered dose, release kinetics, pharmacological activity, safety, and manufacturing reproducibility under application-relevant conditions. Such evidence will help define when architectural control provides a measurable formulation advantage and when structural simplification or redesign is required.

Future development should therefore focus on quantitative structure–performance relationships linking critical structural attributes with delivered dose, intracellular availability or local exposure, release kinetics, pharmacological activity, safety, and batch reproducibility. Translation will further require standardized characterization of structural heterogeneity, storage and processing stability, scalable manufacturing, and context-specific pharmacokinetic and regulatory evaluation [25,45,123,156]. Establishing these relationships will clarify when architectural control provides a measurable pharmaceutical advantage and support the rational development of nucleic acid systems for drug delivery, molecular diagnostics, regenerative biomaterials, vaccination, and related biomedical applications.

## Data Availability

Restrictions apply to the availability of these data. Data were obtained from a third party and are available from the authors with the permission of the third party.
